# Genetic trade‐offs between complex diseases and longevity

**DOI:** 10.1111/acel.13654

**Published:** 2022-06-26

**Authors:** Dingxue Hu, Yan Li, Detao Zhang, Jiahong Ding, Zijun Song, Junxia Min, Yi Zeng, Chao Nie

**Affiliations:** ^1^ College of Life Sciences University of Chinese Academy of Sciences Beijing China; ^2^ BGI‐Shenzhen Shenzhen China; ^3^ The First Affiliated Hospital, Institute of Translational Medicine, School of Medicine Zhejiang University Hangzhou China; ^4^ Center for Healthy Aging and Development Studies, National School of Development Peking University Beijing China; ^5^ Center for the Study of Aging and Human Development and Geriatrics Division Medical School of Duke University Durham North Carolina USA

**Keywords:** complex disease, genetics, human longevity, polygenic risk score, trade‐offs

## Abstract

Longevity was influenced by many complex diseases and traits. However, the relationships between human longevity and genetic risks of complex diseases were not broadly studied. Here, we constructed polygenic risk scores (PRSs) for 225 complex diseases/traits and evaluated their relationships with human longevity in a cohort with 2178 centenarians and 2299 middle‐aged individuals. Lower genetic risks of stroke and hypotension were observed in centenarians, while higher genetic risks of schizophrenia (SCZ) and type 2 diabetes (T2D) were detected in long‐lived individuals. We further stratified PRSs into cell‐type groups and significance‐level groups. The results showed that the immune component of SCZ genetic risk was positively linked to longevity, and the renal component of T2D genetic risk was the most deleterious. Additionally, SNPs with very small *p*‐values (*p* ≤ 1x10^‐5^) for SCZ and T2D were negatively correlated with longevity. While for the less significant SNPs (1x10^‐5^ < *p* ≤ 0.05), their effects on disease and longevity were positively correlated. Overall, we identified genetically informed positive and negative factors for human longevity, gained more insights on the accumulation of disease risk alleles during evolution, and provided evidence for the theory of genetic trade‐offs between complex diseases and longevity.

## Graphical abstract

Lower genetic risks of stroke and hypotension were observed in centenarians, while higher genetic risks of schizophrenia (SCZ) and type 2 diabetes (T2D) were detected in long‐lived individuals. The immune component of SCZ genetic risk was positively linked to longevity, and the renal component of T2D genetic risk was the most deleterious. For variants that most significant in SCZ decreases lifespan whereas those less significant variants increases lifespan.

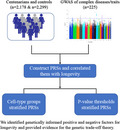

## 1 INTRODUCTION

Human longevity is influenced by many complex diseases and lifestyles. Stroke and ischemic heart disease were the leading causes of death (Zhou et al., 2019). Diabetes (Franco et al., 2007), cardiovascular diseases (Franco et al., 2007), and body mass index (BMI) (Abdelaal et al., 2017) have been reported to be associated with higher mortality. Healthy lifestyles, such as consuming whole grain foods (Hu et al., 2020), physical exercises (Garatachea et al., 2015; Li et al., 2020), and calorie restriction (Hwangbo et al., 2020) are beneficial for promoting healthy aging. These correlations between longevity and complex diseases/traits may be ascribed to shared genetic components. Negative genetic correlations were found between longevity and cardiovascular diseases, smoking, type 2 diabetes (T2D) as well as Alzheimer's disease (Broer et al., 2015; Gutman et al., 2020; McDaid et al., 2017; Nebel et al., 2011; Tesi et al., 2020; Timmers et al., 2020). Positive genetic associations were identified for education and exercise (McDaid et al., 2017). Genome‐wide association study (GWAS) of lifespan and human longevity also identified many pleiotropic genes. *APOE* is the most replicated longevity‐related gene (Broer et al., 2015; Deelen et al., 2014; Deelen et al., 2019; Joshi et al., 2017; Nebel et al., 2011; Sebastiani et al., 2017), and it is also a well‐known gene associated with Alzheimer's disease (Schachter et al., 1994). An allele of the *PON1* (*Paraoxonase 1*) gene has been linked to a higher risk of cardiovascular diseases and also underrepresented in centenarians (Bhattacharyya et al., 2008). All the above reports suggested that pleiotropy is a common event in longevity and complex diseases/traits (Fernandes et al., 2016).

It is not always the case that increased disease genetic risks were linked to higher mortality. It is reported that the number of disease risk alleles, including those of coronary artery disease (CAD), heart failure, cancer, and T2D, was not reduced in long‐lived individuals compared with that in middle‐aged people (Beekman et al., 2010; Erikson et al., 2016; Revelas et al., 2019). Consistent evidence showed that CAD had a negative correlation with longevity (Deelen et al., 2019; Erikson et al., 2016; McDaid et al., 2017). However, conflicting correlational evidence was reported between longevity and T2D (Beekman et al., 2010; Deelen et al., 2019; Erikson et al., 2016; McDaid et al., 2017). Recently, one study showed that most risk SNPs of the Alzheimer's disease (AD) associated with decreased odds of longevity, but some SNPs increased the probability of both AD and longevity (Tesi et al., 2021).

Although some genetic correlations between complex diseases and longevity have been studied, there are still many unproven associations. Therefore, we performed systematic analyses between genetic risks of complex diseases and longevity. We generated polygenic risk scores (PRSs) of 225 complex diseases/traits for 2178 centenarians and 2299 middle‐aged individuals from the Chinese Longitudinal Healthy Longevity Survey cohort (CLHLS). Each PRS was used to predict whether a person is a centenarian to study the relationships between genetic risks of complex diseases/traits and longevity. We further partitioned the SNPs into cell‐type groups and different *p*‐value groups. Next, we annotated the pleiotropic SNPs into genes and gene ontology (GO) terms to gain more functional information about the pleiotropic genes. Finally, all the PRSs were put into one model to predict longevity and to evaluate how much proportion of genetics of complex diseases/traits could contribute to longevity.

## 2 RESULTS

### 2.1 Summary of study dataset

To evaluate the genetic correlations between longevity and complex diseases/traits, we have constructed 225 PRSs of complex diseases/traits, based on well‐selected GWAS meta‐analysis summary statistics of complex diseases/traits, to predict longevity. All the summary statistics covered a wide range of phenotypes, which can be classified into 9 distinct categories, including mental disorders (*n* = 17), age‐related complex diseases (*n* = 2), cardiovascular diseases and related factors (*n* = 9), type 2 diabetes and related traits (*n* = 12), other complex diseases (*n* = 8), anthropometrics (*n* = 18), metabolic indexes (*n* = 141), body compositions (*n* = 13), and social lifestyles (*n* = 5). The detailed characteristics of the phenotypes and sources were described in Table S1. 2178 centenarians and 2299 middle‐aged controls (aged 40–59) from CLHLS (Zeng, 2012) cohort were genotyped by Illumina HumanOmniZhongHua‐8 BeadChips (Zeng et al., 2016). 5,594,914 SNPs were retained after quality control (QC).

### 2.2 Both positive and negative correlations between PRSs and longevity were identified among multiple complex traits

**FIGURE 1:**
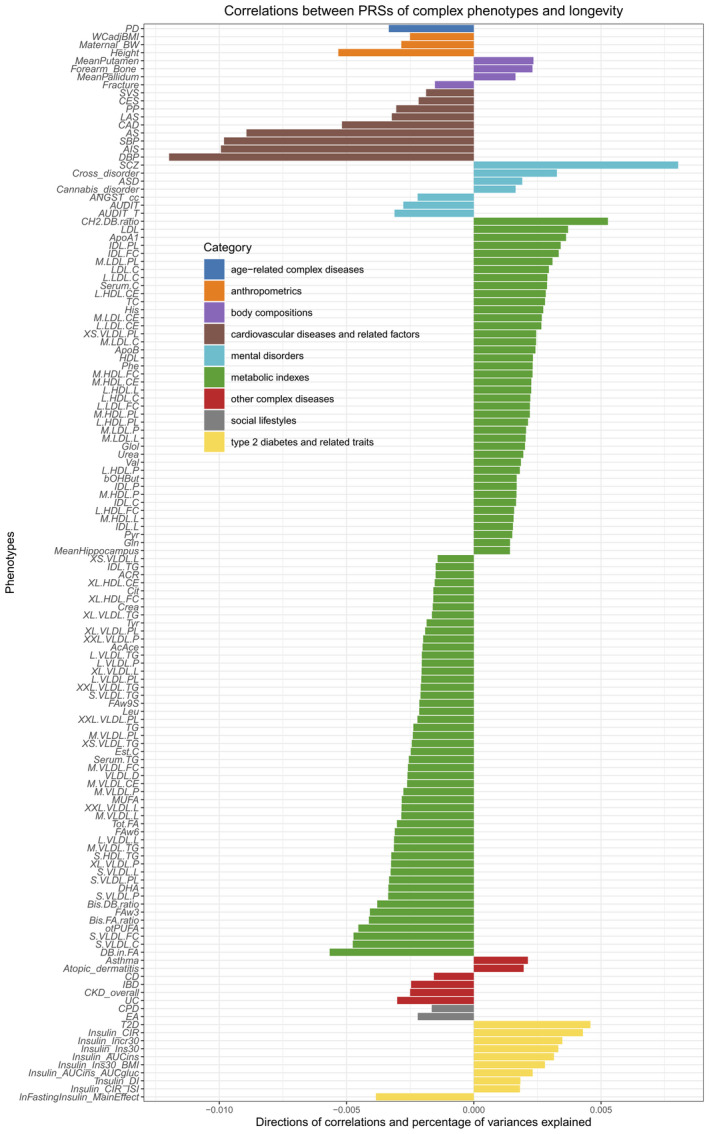
134 PRSs of complex traits could predict longevity significantly. The length of the bar represents the proportion of longevity explained by PRS. The minus sign indicates negative correlation. Phenotype abbreviations were given in Table S1

SNPs for the construction of PRSs were selected through thresholding *p*‐values. The effects of selected SNPs from GWAS summary statistics were used as weights to sum the SNP genotypes. Multiple PRSs of each trait were utilized to predict whether a person is a centenarian or not. The best PRSs for one trait was the one which gained smallest *p*‐value in the predictions. Multiple testing corrections were further conducted in the *p*‐values of best PRSs for complex traits. Overall, we identified 134 PRSs of complex phenotypes correlated with longevity after multiple testing adjustment using false discovery rate (FDR; FDR‐adjusted *p* < 0.05; Figure 1 and Table S2).

Most of the PRSs of clinically diagnosed diseases were negatively correlated with longevity, including those of Parkinson's disease (PD), inflammatory bowel disease (IBD), stroke, CAD, and kidney disease. Inversely, the PRSs of some psychiatric disorders, such as schizophrenia (SCZ), autism spectrum disorder, cannabis disorder, insulin‐related traits, and atopic dermatitis, were positively correlated with longevity. In the PRSs of body measurements, the direct measures such as weight, hip circumference, and waist circumference were not correlated with longevity. While being integrated into indexing traits, some of the size measures were correlated with longevity. For instance, waist circumference adjusted by BMI as a strong predictor of harmful intra‐abdominal fat mass (Berentzen et al., 2012) was negatively correlated with longevity. The PRS of height was negatively correlated with longevity; it was consistent with the result of a trans‐ethnical study (Sakaue et al., 2020). In terms of brain and bone‐related measures, the PRS of fracture was negatively correlated with longevity, while the PRSs of forearm bone mineral density, the intracranial volume of putamen, and pallidum of brain were positively correlated with longevity.

Interestingly, among the metabolic measures, “good lipids” and “bad lipids” were identified. Generally, the PRSs of very large and extreme large very‐low‐density lipoproteins (VLDL) were negatively correlated with longevity, while the PRSs of medium and large high‐density lipoproteins (HDL) were positively correlated with longevity. VLDL level is one of risk factors for atherosclerotic cardiovascular disease (Prenner et al., 2014; Varbo et al., 2013). Epidemiological evidence suggested that higher HDL levels may serve a protective role from numerous age‐related diseases (Milman et al., 2014; Wang et al., 2018). The PRS of the average number of double bonds in fatty acids (DB.in.FA) was significantly negatively correlated with longevity, while the PRS of the average number of methylene groups per double bond (CH2.DB.ratio) was positively influencing longevity. The DB.in.FA has been found negatively correlated with longevity in *C. elegans* (Shmookler Reis et al., 2011 ), the double bonds number and methylene in a fatty acid may be related to oxidative phosphorylation (Parvez et al., 2018; Valencak & Azzu, 2014).

After Bonferroni correction, there were still 16 PRSs could significantly predict longevity (*p* < 2.22 × 10^−4^; Figure S1 and Table 1). The top longevity related PRSs including SCZ, T2D and its related traits, CAD, stroke (any stroke and any ischemic stroke), metabolic traits (blood lipids and related ratios), and height. The PRS of diastolic blood pressure (DBP) could explain the highest proportion of variation for longevity (*R* ^2^ = 0.012).

**Table 1:** Correlations between PRSs of complex traits and longevity

Phenotype	Threshold	PRS.R2	Effect	Num_SNPs	*p*	Categories
DBP	1.18E‐02	1.20E‐02	Negative	17,121	2.79E‐10	Cardiovascular diseases and related factors
AIS	5.00E‐08	9.93E‐03	Negative	18	8.74E‐09	Cardiovascular diseases and related factors
SBP	6.95E‐03	9.82E‐03	Negative	14,457	1.07E‐08	Cardiovascular diseases and related factors
AS	5.00E‐08	8.93E‐03	Negative	15	4.82E‐08	Cardiovascular diseases and related factors
SCZ	7.69E‐01	8.03E‐03	Positive	111,079	2.28E‐07	Mental disorders
DB.in.FA	5.00E‐08	5.66E‐03	Negative	9	1.34E‐05	Metabolic indexes
Height	3.50E‐02	5.32E‐03	Negative	20,416	2.46E‐05	Anthropometrics
CH2.DB.ratio	5.00E‐08	5.27E‐03	Positive	13	2.68E‐05	Metabolic indexes
CAD	2.65E‐03	5.18E‐03	Negative	1860	3.15E‐05	Cardiovascular diseases and related factors
S.VLDL.C	5.00E‐08	4.75E‐03	Negative	30	6.69E‐05	Metabolic indexes
S.VLDL.FC	5.00E‐08	4.73E‐03	Negative	27	7.03E‐05	Metabolic indexes
T2D	6.70E‐01	4.58E‐03	Positive	69,656	9.07E‐05	Type 2 diabetes and related traits
otPUFA	5.00E‐08	4.54E‐03	Negative	26	9.75E‐05	Metabolic indexes
Insulin_CIR	3.04E‐02	4.29E‐03	Positive	6737	1.51E‐04	Type 2 diabetes and related traits
Bis.FA.ratio	5.00E‐08	4.12E‐03	Negative	12	2.02E‐04	Metabolic indexes
FAw3	5.00E‐08	4.08E‐03	Negative	9	2.18E‐04	Metabolic indexes

Phenotype: the names of the complex diseases/traits; Threshold: best *p*‐value threshold; PRS.R2: variance explained by the PRS; Effect: the impact of genetic risk of complex diseases on longevity; Num_SNPs: the number of the SNPs in PRS construction; *p*: *p*‐value of the model fit; Categories: the category of the complex diseases/traits. Phenotype abbreviations were given in Table S1.

### 2.3 PRSs of complex diseases/traits were associated with longevity while masking *APOE* region

Among the top 16 PRSs that were significantly correlated with longevity, there were many complex diseases/traits associated with *APOE*. For example, the role of Apolipoprotein E in lipid metabolism has been well established (Dose et al., 2016; Mahley, 2016), *APOE* was also reported to be associated with the risk of cardiovascular diseases and diabetes mellitus (Eichner et al., 2002), and *APOE* is the most replicated longevity‐related gene (Broer et al., 2015; Deelen et al., 2014; Deelen et al., 2019; Joshi et al., 2017; Nebel et al., 2011; Sebastiani et al., 2017). In order to see whether the associations between longevity and the 16 PRSs of complex diseases/traits were dominated by *APOE* or contributed by multiple genetic factors, we excluded the *APOE* region to construct PRSs for the 16 complex diseases/traits and evaluated their relationships with longevity.

We defined the *APOE* region by LDBlockShow (Dong et al., 2021) in our genotype dataset that all individuals are Han Chinese, and replicated it using the genotypic data of Eastern Asian of the 1000 Genome Project. The results of centenarians and controls showed that 8 LD blocks were in the *APOE* region, as SNPs located from position 45,361,224 to 45,432,557 base pairs on chromosome 19 (Figure S2a). The result of Eastern Asian of the 1000 Genome Project showed that 14 LD blocks were in the *APOE* region, from position 45,361,224 to 45,436,657 base pairs on chromosome 19 (Figure S2b). The *APOE* region was largely overlapped in our cohort and the Eastern Asian population. Finally, we excluded the larger *APOE* region of chr19:45,361,224‐45,436,657 from the GWAS summary statistics of the 16 complex diseases/traits to construct PRSs and correlated them with longevity. The results showed that PRSs of complex diseases/traits were still associated with longevity while masking *APOE* region (Table S3). After Bonferroni correction for all the PRSs of 225 complex diseases/traits, including DBP, any ischemic stroke (AIS), systolic blood pressure (SBP), any stroke (AS), schizophrenia, height, T2D, other polyunsaturated fatty acids than 18:2 (otPUFA), DB.in.FA, corrected insulin response (Insulin_CIR), ratio of bis‐allylic groups to total fatty acids in lipids (Bis.FA.ratio), omega‐3 fatty acids were still significantly correlated with longevity with *p*‐value < 2.22 × 10^−4^ (*p* < 0.05/225). The *p*‐values of CH2.DB.ratio, free cholesterol in small very‐low‐density lipoprotein (S.VLDL.FC), and total cholesterol in small very‐low‐density lipoprotein (S.VLDL.C) became larger than 2.22 × 10^−4^, but still correlated with longevity with the *p*‐value = 2.82 × 10^−4^ of CH2.DB.ratio, *p*‐value = 4.23 × 10^−4^ of S.VLDL.FC, and *p*‐value = 5.62 × 10^−4^ of S.VLDL.C.

### 2.4 Cell‐type group‐specific PRSs show different directions of correlations with human longevity

The above analyses showed the differences of genetic components for complex traits contributing to longevity. Then, we further studied that within a complex trait, how do disproportionated genetic contributions of functional categories influence longevity. The SNPs were annotated into 220 cell types, and cell‐type annotations were combined into 10 groups representing biological systems for human. Cell‐type specific PRSs and cell‐type group‐specific PRSs were generated. We assessed statistical significance at *p* < 0.05 after FDR corrections for 220 × 16 = 3520 tests, the numbers represent 220 cell types and top 16 complex phenotypes whose PRSs were most significantly associated with longevity.

**FIGURE 2:**
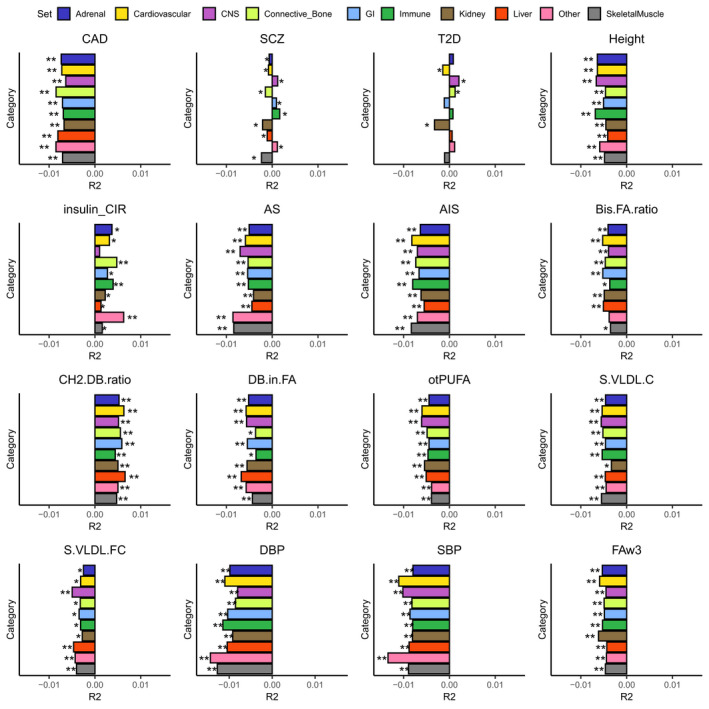
Correlations between cell‐type group‐specific PRSs of complex traits and longevity. The length of the bar represents the proportion of longevity explained by cell‐type group‐specific PRS. The minus sign indicates negative correlation. Phenotype abbreviations were given in Table S1. *FDR‐adjusted *p* < 0.05. **p < 0.05 after Bonferroni correction

Cell‐type group‐specific PRS results for the 16 traits mentioned above were shown in Figure 2. Most of the complex diseases/traits showed negative correlations with longevity at all cell‐type groups, including CAD, height, stroke, DBP and SBP, Bis.FA.ratio, DB.in.FA, otPUFA, S.VLDL.C and S.VLDL.FC. Two traits, Insulin_CIR and CH2.DB.ratio, showed positive correlations with longevity at all cell‐type groups. SCZ and T2D showed bi‐directional correlations. For SCZ, all cell‐type group‐specific PRSs could predict longevity significantly (FDR‐adjusted *p* < 0.05). PRSs of central nervous system (CNS), gastrointestinal, immune groups were positively correlated with longevity, while that of adrenal, cardiovascular, connective or bone, kidney, liver, and skeletal muscle cell‐type groups were negatively correlated with longevity. The PRS of the immune cell‐type group explained the highest proportion of variation for longevity in positive correlation. Existing literature revealed that the hyperactive immune system was correlated with SCZ (Khandaker et al., 2015; Müller & Schwarz, 2010), and the enhanced immune system may be an advantage to longevity (Zeng et al., 2016). In T2D, the PRSs for cardiovascular, CNS, connective or bone and kidney groups passed the threshold (FDR‐adjusted *p* < 0.05). PRSs of CNS and connective or bone groups showed positive correlations with longevity, while PRS for cardiovascular and kidney groups showed negative correlations with longevity. T2D is a major risk factor of kidney disease, and renal disease is an important complication (Tancredi et al., 2015), our results indicated that T2D‐associated kidney diseases may be a great risk factor of mortality. The significant cell‐type specific PRS results for the 16 traits were displayed in Table S4. Most of the complex traits correlated with brain structure‐related and immunity‐related cell types.

Then, we excluded the *APOE* region chr19:45,361,224‐45, 436,657 from the GWAS summary statistics of the 16 complex diseases/traits to construct cell‐type group‐specific PRSs and correlated them with longevity. Most of our reported associations were still significant after masking the *APOE* region (Figure S3). Cell‐type group‐specific PRSs of S.VLDL.FC and S.VLDL.C could explain smaller proportion of the longevity.

### 2.5 Dissecting effects of SNPs in SCZ, T2D, and longevity

**FIGURE 3:**
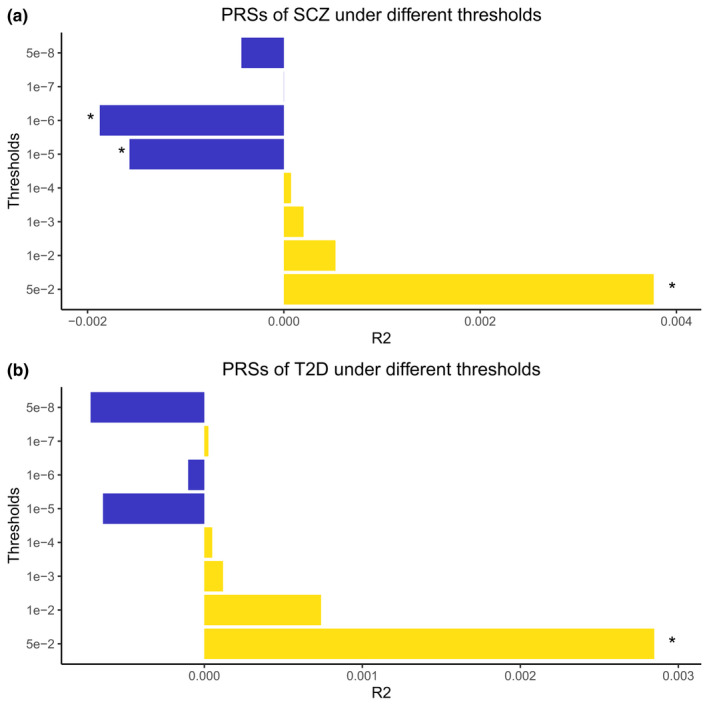
Directions of correlations and percentage of variances explained by PRSs in different thresholds. The length of the bar represents the proportion of longevity explained by PRS. The minus sign indicates negative correlation. *p < 0.05

The above analyses suggested that SNPs across the genome have different directions of effects between complex diseases and longevity, especially for SCZ and T2D. In order to deeply explore the effects of pleiotropic SNPs on longevity and complex diseases, we detailed stratified PRSs using different *p*‐value thresholds in SCZ and T2D. We identified similar patterns in SCZ and T2D. For the most significant groups of SNPs in disease summary statistics, the increased disease risks were associated with a reduced chance of becoming centenarian. While for those SNPs with less significances, the increased disease risks also increase the chance of being long‐lived (Figure 3a, b).

**FIGURE 4:**
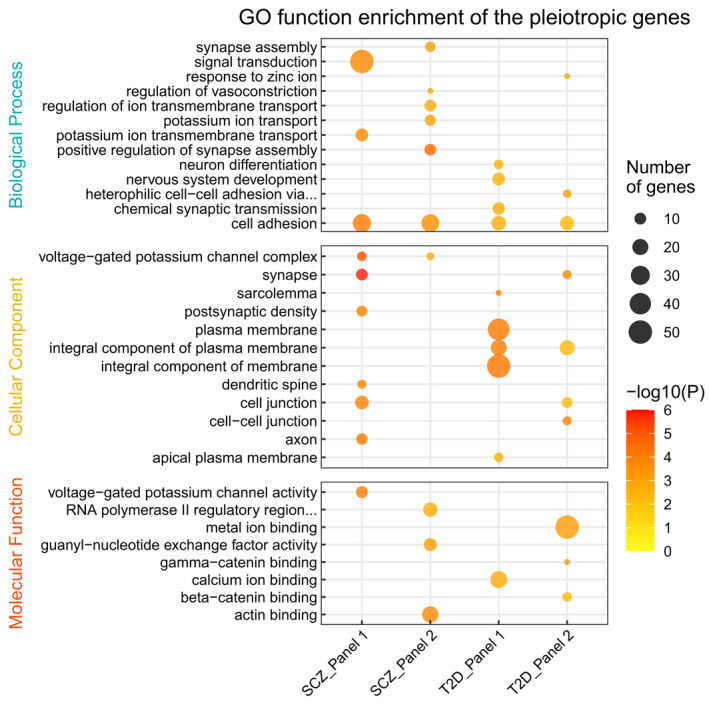
GO enrichment of the pleiotropic genes for SCZ and T2D. SCZ_Panel 1: Genes both increasing chance of SCZ and longevity; SCZ_Panel 2: Genes increasing SCZ risk and reducing the chance of longevity; T2D_Panel 1: Genes both increasing chance of T2D and longevity; T2D_Panel 2: Genes increasing T2D risk and reducing the chance of longevity

We were then interested in searching for the functions of pleiotropic SNPs/Genes in longevity and SCZ/T2D, especially those increasing both disease risks and probability of being centenarian. In order to do this, two pairs of summary statistics were compared, SCZ vs. longevity and T2D vs. longevity. Within each pair, the effects of SNPs with nominal significance (*p* < 0.05) in both phenotypes were selected and compared (Figure S4a,b). All the compared SNPs could be classified into two categories: (1) both increasing chance of diseases and longevity (panel 1); (2) increasing disease risks and reduce life expectancy (panel 2). The top 10 significant GO terms for two panels of genes were shown in Figure 4. Only two terms were overlapped between panels 1 and 2, for SCZ and T2D, respectively. This suggesting that the functions of panels 1 and 2 were complementary.

### 2.6 Comparing the effects of longevity‐related genes in SCZ and T2D

**FIGURE 5:**
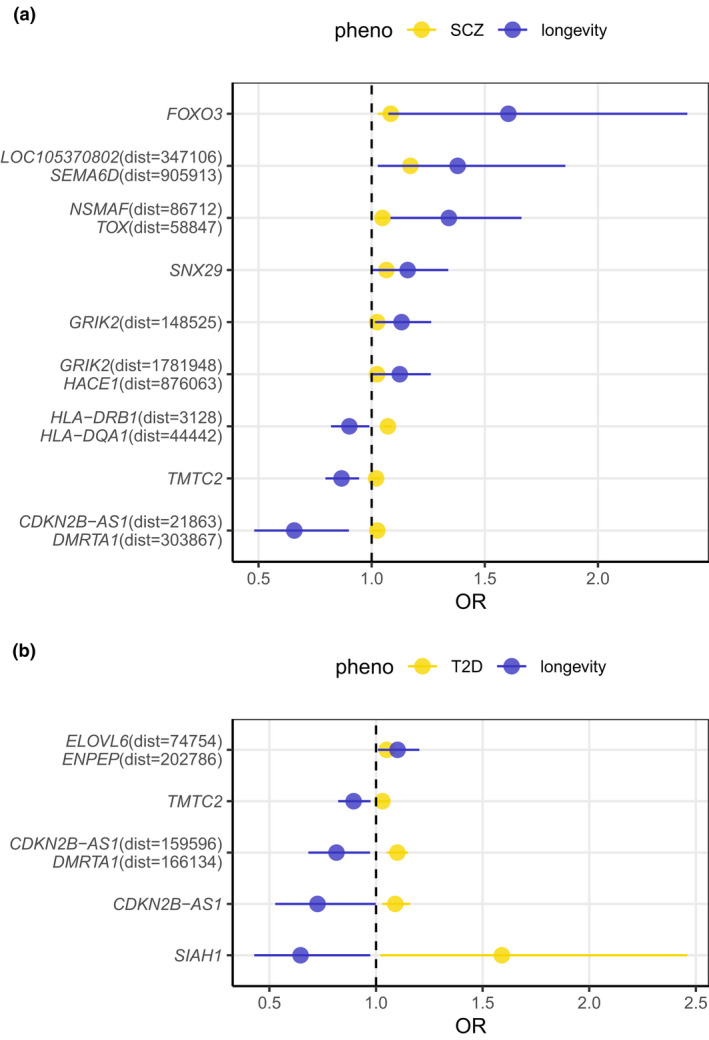
Effect sizes of longevity‐related genes in SCZ and T2D. (a) Effects of longevity‐related genes in SCZ; (b) effects of longevity‐related genes in T2D. Dist: The distant from the SNP to the gene

We evaluated longevity‐related SNPs/Genes from the largest meta‐analysis of longevity (Deelen et al., 2019). The information of SNPs/Genes was shown in Table S5. Effect size of these genes in SCZ and T2D was compared to their effects of longevity (Figure 5a, b, and Table 2). There were 9 longevity‐related SNPs have nominally significant (*p* < 0.05) effects on SCZ. Among these SNPs, 6 SNPs showed positive effects on both longevity and SCZ, 3 SNPs showed opposite effects on longevity and SCZ. 5 longevity‐related SNPs showed nominally significant (*p* < 0.05) effects on T2D, 1 SNP showed both positive effects on longevity and T2D, the others showed opposite effects on longevity and T2D. *FOXO3* was a famous longevity‐related gene (Broer et al., 2015; Tanaka et al., 2017; Timmers et al., 2019). The allele T of rs72942514 within *FOXO3* was nominally significantly associated with longevity (OR = 1.604, *p* < 0.05), and it was also correlated with higher risk of SCZ (OR = 1.084, *p* < 0.005; Figure 5a, Table 2). The SNP which had both positive effects on longevity and T2D located on intergenic region.

**Table 2:** Effect size of longevity‐related SNPs in schizophrenia and type 2 diabetes

Phenotype	Gene	SNP	CHR	BP	A1	A2	OR_dis (CI 95%)	P_dis	OR_long (CI 95%)	P_long
SCZ	*SNX29*	rs118115187	16	12,385,672	G	A	1.065 (1.011–1.119)	0.021	1.159 (1.004–1.338)	0.044
SCZ	*LOC105370802*(dist =347,106), *SEMA6D*(dist = 905,913)	rs149065260	15	46,570,373	T	C	1.171 (1.046–1.296)	0.014	1.380 (1.027–1.856)	0.033
SCZ	*TMTC2*	rs2403035	12	83,282,718	A	C	1.020 (1.001–1.038)	0.037	0.867 (0.796–0.944)	0.001
SCZ	*GRIK2*(dist = 1,781,948), *HACE1*(dist = 876,063)	rs35574272	6	104,299,906	G	A	1.024 (1.004–1.044)	0.022	1.124 (1.001–1.261)	0.047
SCZ	*NSMAF*(dist = 86,712), *TOX*(dist = 58,847)	rs72649409	8	59,659,124	T	C	1.048 (1.024–1.072)	0.000	1.341 (1.083–1.662)	0.007
SCZ	*FOXO3*	rs72942514	6	108,928,380	T	C	1.084 (1.027–1.141)	0.005	1.604 (1.074–2.395)	0.021
SCZ	*GRIK2*(dist = 148,525)	rs72957936	6	102,666,483	A	G	1.024 (1.001–1.047)	0.048	1.132 (1.015–1.263)	0.026
SCZ	*CDKN2B‐AS1*(dist = 21,863), *DMRTA1*(dist = 303,867)	rs7859532	9	22,142,956	C	A	1.025 (1.002–1.048)	0.034	0.658 (0.481–0.899)	0.009
SCZ	*HLA‐DRB1*(dist = 3128), *HLA‐DQA1*(dist = 44,442)	rs9270560	6	32,560,741	T	C	1.072 (1.053–1.091)	0.000	0.901 (0.821–0.990)	0.029
T2D	*TMTC2*	rs10862520	12	83,293,097	A	G	1.030 (1.000–1.060)	0.046	0.895 (0.822–0.974)	0.010
T2D	*ELOVL6*(dist = 74,754), *ENPEP*(dist = 202,786)	rs11728821	4	111,194,525	G	A	1.050 (1.020–1.080)	0.001	1.101 (1.009–1.203)	0.032
T2D	*CDKN2B‐AS1*	rs17694555	9	22,051,295	G	A	1.090 (1.030–1.160)	0.002	0.726 (0.527–0.998)	0.049
T2D	*CDKN2B‐AS1*(dist = 159,596), *DMRTA1*(dist = 166,134)	rs1887268	9	22,280,689	C	T	1.100 (1.050–1.150)	0.000	0.815 (0.683–0.972)	0.023
T2D	*SIAH1*	rs34791504	16	48,401,791	T	C	1.590 (1.020–2.460)	0.040	0.646 (0.429–0.973)	0.036

dist: distant of the SNP to the gene. CHR: Chromosome; BP: the base position, based on Genome Reference Consortium Human Build 37 (GRCh37); A1: risk allele; OR_dis: odds ratio of complex diseases, (i.e., odds to develop complex diseases when carrying the effect allele); P_dis: *p*‐values in complex disease GWAS; OR_long: odds ratio of longevity, (i.e., odds to become a centenarian when carrying the effect allele): P_long: *p*‐values in longevity GWAS; The rsID is based on dbSNP build 150, dist: means the distant from the SNP to the gene.

### 2.7 Estimating the contribution of all PRSs

**FIGURE 6:**
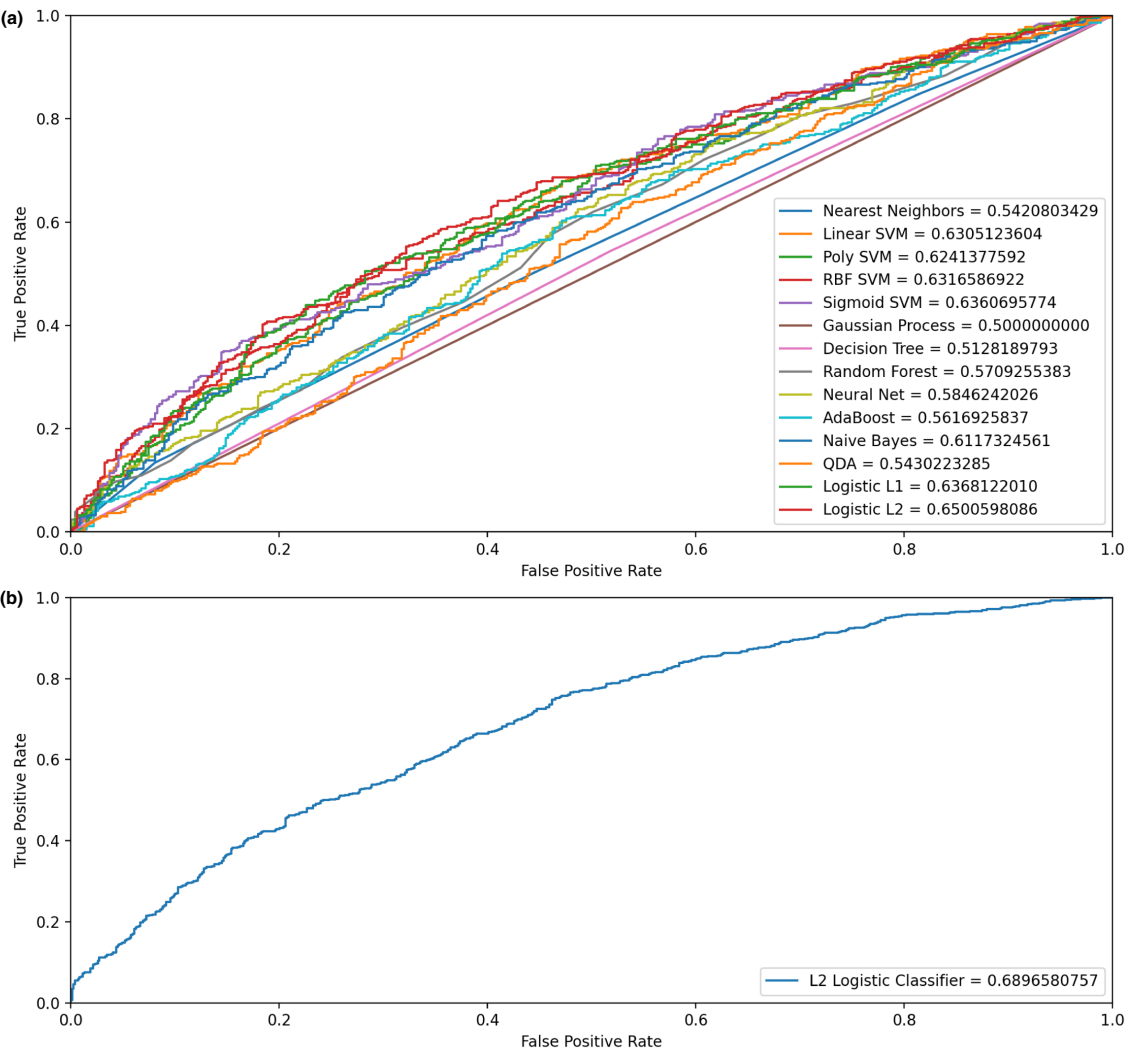
Using all PRSs of complex phenotypes to predict longevity. (1) Comparisons of the prediction efficiency of different methods. (2) The optimized prediction from the logistic regression model

The above analyses were performed using a single PRS to predict longevity. Next, we were interested in using all PRSs of complex diseases/traits to predict longevity and evaluate the overall contribution of all PRSs to longevity. To find an optimized regression method, we trained our data with multiple common classifiers, including SVM based classifier, KNN classifier, Naive Bayes classifier, logistic regression, Decision Tree, and Random Forest classifier. 10‐fold cross‐validation was conducted for model construction, and 100 iterations with randomly split training and validation sets were run to avoid overfitting. Consequently, the logistic regression classifier provided the best prediction (Figure 6a). Further optimizing parameters of logistic regression could achieve AUC = 0.69 and pseudo‐*R* ^2^ = 0.08 (Figure 6b), indicating that all PRSs together could only explain a small proportion of the variance of longevity. The coefficient of each PRSs in the best prediction model was shown in Table S6.

## 3 DISCUSSION

One might expect that the genetic risks of complex diseases would be lower in long‐lived people as compared to that in younger controls. In this study, we provided evidence in support of this expectation, most of the PRSs of clinically diagnosed diseases were negatively correlated with longevity, including PD, IBD, stroke, CAD, and kidney disease. Previous studies also provide sufficient evidence to support this expectation, reduced risks for height (Sakaue et al., 2020), CAD and its related traits (Timmers et al., 2019) such as DBP (Sakaue et al., 2020), SBP (Sakaue et al., 2020) contributed to longevity or lifespan (Broer et al., 2015; Deelen et al., 2019; Sakaue et al., 2020; Timmers et al., 2019).

However, relationships between longevity and complex diseases were not always as expected, in which increased disease risks were not necessarily linked to higher mortality. We identified that the PRSs of some psychiatric disorders, such as SCZ, autism spectrum disorder (ASD), cannabis disorder, and atopic dermatitis, were positively correlated with longevity, as well as T2D. We have further investigated these intriguing associations and possible underlying mechanisms by stratified PRS analyses. The results showed that most of the SNPs with very small *p*‐values (*p* ≤ 1 × 10^−5^) for SCZ/T2D were negatively correlated with longevity. While for the less significant SNPs (1 × 10^−5^ < *p* ≤ 0.05), their effects on disease and longevity were positive. It was reported that the cancer incidence in first‐degree relatives of patients with SCZ had significantly decreased risks of overall cancers (Catts et al., 2008), and this finding has been replicated in an independent study (Ji et al., 2013). Many CNS disorders have inversed cancer comorbidity (Tabares‐Seisdedos & Rubenstein, 2013). Further, in the term of cell‐type levels, PRS of the immune cell‐type group explained the highest proportion of variation in the positive correlations. The hyperactive immune system‐related genes may be causes of SCZ (Comer et al., 2020). Meanwhile, the enhanced immune system could be beneficial for an increasing long‐life span (Pinti et al., 2016). If detailed looking at gene level effects, *FOXO3* gene was a famous gene for human longevity, which controls the magnitude of T‐cell immune response by modulating dendritic cell function. The enhanced capacity of Foxo3‐deficient dendritic cells to sustain T‐cell viability by producing increased amounts of interleukin 6 (IL‐6) (Dejean et al., 2009). The increased expression of IL‐6 would enhance cell survival and transform cell growth in human malignant cholangiocytes (Meng et al., 2006). Also, the decreased expression of *FOXO3A* was found in acute SCZ patients (Gu et al., 2021). It is possible that the reduced expression of *FOXO3* leads to enhanced IL‐6 expression and formed an enhanced immune system. In T2D cell‐type group‐specific PRSs, PRS of kidney‐related cell‐type group explained the highest proportion of variation for longevity. T2D is a major risk factor of kidney disease, and renal disease is an important complication causing mortality (Shmookler Reis et al., 2011). Still, some genetic factors were contributing to the positive associations between T2D genetic risk and longevity, especially for those genes in the CNS. Glucose is the main source of energy for brain and brain consumes ~20% of glucose‐derived energy to maintain the neuronal activities. It is reported that glucose acts as a double edge sword in regulating the functions of SIRT1 (Chattopadhyay et al., 2020). SIRT1 has long been known to be a longevity factor. Glucose binds and modifies SIRT1 ultimately reduces its levels. Loss of SIRT1 is associated with obesity and aging. On the contrary, overactivation of the longevity factor SIRT1 was also detrimental to liver physiology and resulted in increased blood glucose levels leading to a pre‐diabetic like state (Chattopadhyay et al., 2020). In this case, a high blood glucose level is associated with high SIRT1.

Our results of the associations between genetic risks of complex diseases and longevity were conflicted with the phenotypic associations. Both SCZ and T2D were reported to increase mortality in many studies (Bardenheier et al., 2016; Hennekens, 2007; Kilbourne et al., 2009; Sikdar et al., 2010). One possible explanation would be that the phenotypes were influenced by genetics, environments, and their interactions. In our prediction model, all the PRSs could only explain a small proportion of the variances of longevity. There were much more effects depend on other factors. The genetic effects of phenotypes may vary between different environments. Our results showed that the immune component of SCZ genetics was beneficial to longevity. While SCZ could also be triggered by many environmental factors, such as early hazards causing fetal growth retardation or drug abuse (Dean & Murray, 2005), these environmental factors may disturb the development of the immune system which may be harmful for health and cause early death. Similar to T2D, air pollution, diet, and physical activity (Dendup et al., 2018) were strongly correlated with T2D. An unhealthy diet would damage renal function in patients with diabetes (Lin et al., 2020). Our results showed that the renal component for genetic risks of T2D significantly reduced the possibility of longevity. Furthermore, the SNP genotypes will not change during one's life, but their impact on vulnerability to mortality could be changed by epigenetics. Age‐related DNA methylation patterns have been reported a lot (Bell et al., 2019; Gensous et al., 2019). Different sets of genes may be activated in response to different age‐ and population‐specific environments and exposures (Ukraintseva et al., 2016).

It is reasonable that some risk genes for diseases are positively related to longevity from the aspect of evolution (Carter & Nguyen, 2011). During the historical process of natural selection, the beneficial mutant was accumulated while the deleterious mutation would be eliminated. Therefore, the existing common variants increasing the disease risks may potentially be protective against some extreme environment.

All these complex G × G and G × E interactions made the genetic effects on longevity highly conditional. In different stages of one's life cycle, distinct environments with diverse lifestyles would all lead to different effects from the same set of genes. This may be the reason why the results of longevity GWAS studies were very hard to be replicated (Broer et al., 2015; Deelen et al., 2014; Deelen et al., 2019; Erikson et al., 2016; Joshi et al., 2017; Nebel et al., 2011; Pilling et al., 2017; Timmers et al., 2019).

The antagonistic pleiotropy effects had been proposed in many articles (Aidoo et al., 2002; Byars & Voskarides, 2020; Carter & Nguyen, 2011; Sørensen et al., 1999; Ukraintseva et al., 2016), but most of them are literature reviews. Our study used a data‐driven approach and constructed PRSs for a wide range of complex phenotypes in the same group of people and compared their effects on longevity. Overall, our results suggested that “risk” or “beneficial” of a common genetic variant is conditional regarding its role in human aging, health, and lifespan. Studying these conditions is crucial for a detailed understand of the aging process, and also essential for personalized medicine which emphasizes the uniqueness of each individual.

In the PRS construction, we choose a clumping and thresholding method (Choi & O'Reilly, 2019). The *p*‐value thresholds were selected in logistic regression model which predicting longevity phenotype. We believe *p*‐value thresholding method is better than the fixed *p*‐value threshold method. Because it is unfair to choose fixed thresholds for different complex phenotypes due to the inconsistent sample sizes of different GWASs, it is uncertain which fixed threshold is the best for PRS construction. In addition, the objective of this study was evaluating the associations between PRSs of complex phenotypes and longevity. This approach can try more possibility of the *p*‐value thresholds and select the best one that could predict longevity phenotype. Therefore, using this approach, we could identify more complex phenotypes whose PRSs could significantly correlated with longevity.

In conclusion, our study evaluated the relationships between PRSs of complex diseases/traits and longevity. We confirmed the genetic risks of most fatal diseases would decrease the chance of being long‐lived. Moreover, we also identified several traits, whose genetic risks may have benefits for longevity. Our study provided evidence for the genetic trade‐off theory. We emphasized the positive effects of disease risk alleles on longevity, which could help explain the origin of diseases genetic components.

## 4 METHODS

### 4.1 Study populations, genotyping, and imputation

Our study included 2178 centenarians (mean age 102.7 ± 3.49 [SD]) and 2299 middle‐aged controls (mean age 48.4 ± 7.44 [SD]) (Zeng et al., 2016). The data were obtained from the Chinese Longitudinal Healthy Longevity Survey cohort (CLHLS) (Zeng, 2012; Zeng et al., 2016; Zeng et al., 2018; Zhao et al., 2018). All the centenarians and middle‐aged controls were genotyped by the Illumina HumanOmniZhongHua‐8 BeadChips, including 600 k common variants (MAF ≥ 5%), 290 k rare variants (MAF < 5%), and 10 k SNPs existing only among Chinese and other Asian populations. We performed standard GWAS QC and imputation for the data, the detailed steps can see the previously published study (Zeng et al., 2016). Briefly, the data QC included two dimensions, samples, and SNPs. All QC assessments and successive filtering were done using PLINK1.9 (Purcell et al., 2007). Samples with more than 1% missing genotyped SNPs, different genetic sex with the record in the phenotypic database were removed. Samples who have genetic relationship within two degree of relatedness were filtered out. SNPs had high rate of missing genotypes and deviated from the Hardy–Weinberg equilibrium (HWE) test (*p* ≤ 1 × 10^−5^) as well as SNPs on X and Y chromosomes and mitochondria were also removed. Principal component analysis (PCA) was performed using SNPs on autosomal chromosome by PLINK1.9 (Purcell et al., 2007) to investigate population stratification. No clear sub‐cluster was observed. Typical north to south grandaunt was demonstrated by the first principal component. Next, the 1000 Genomes Project integrated phase 1 release was used as reference panel to infer the genotypes of all SNPs (MAF > 1%) by IMPUTE2 (Marchini et al., 2007), imputed SNPs with a quality score less than 0.9 were discarded before analysis. After imputation, we performed SNP QC again as discussed above. Finally, 5,594,914 SNPs were retained and used as target data to construct PRSs.

### 4.2 GWAS summary statistics data resources and preprocessing

Firstly, the GWAS summary statistics collected by LD hub (Zheng et al., 2017), a centralized database of summary‐level GWAS results for complex diseases/traits from different publicly available resources/consortia, were considered. But some of the them were out of date, some of them were conducted by sex‐stratified approaches, and some of the GWAS studies involve multiple GWAS summary statistics. We used the following criteria to filter the GWAS summary statistics: (1) Not sex‐stratified; (2) Multi‐ethnic meta‐analysis was preferred to single‐ethnic research; and (3) For multiple GWASs of the same phenotype, the one with the largest sample size was selected. As a result, 200 of the GWAS summary statistics were collected based on LD hub. 16 of them were updated for obtaining the latest GWAS results, including T2D, asthma, 5 mental disorders, 2 anthropometrics, 3 body composition, and 4 metabolism indexes. In addition, we added 8 cardiovascular‐related diseases and risk factors (stroke and blood pressure‐related complex traits) since cardiovascular diseases were the leading causes of death, and 17 GWAS summary statistics of 2 anthropometrics, 8 body compositions, and 7 metabolic indexes. Therefore, a total of 225 complex diseases/traits were included in this study. The detailed characteristics of the phenotypes and sources of GWAS summary data were described in Table S1.

All the coordinates of SNPs in GWAS summary statistics were converted to the coordinates of hg19/GRCh37 using UCSC LiftOver tool (Kent et al., 2002). Then, the genotypes of each SNPs were matched, and the effect size was converted to ensure the testing allele for all the traits were the same. SNPs with mismatched alleles were flipped into their complementary alleles to match again. SNPs that had different or ambiguous genotypes in multiple studies were excluded. The clean GWAS summary statistics were used as base data to construct PRSs.

### 4.3 Construction of PRSs and prediction of longevity phenotype

According to the effect size and *p‐value*s of SNPs in large‐scale GWAS summary statistics (base data), we constructed PRSs for centenarians and middle‐aged controls using their genotype data using PRSice‐2 (Choi & O'Reilly, 2019). The following formula was used to calculate PRSs. Assuming *S*_*i*_ is the summary statistic of the *i* ^th^ effective allele, *G*_*ij*_ is the number of the *i* ^th^ effective alleles observed in *j* ^th^ individual (0, 1, 2, respectively), *M*_*j*_ is the number of alleles included in the PRS of the *j* ^th^ individual.

$${\mathrm{PRS}}_{j}=\sum_{i}\frac{S_{i}\times G_{ij}}{M_{j}}$$

We derived PRSs of complex diseases for each individual by a clumping and thresholding method. First, linkage disequilibrium (LD) clumping was performed on genotypic data of centenarians and controls using a clumping option of *r* ^2^ > 0.1 and a window of 500 kb. Then, PRSs of complex diseases of individuals were computed by different *p‐value* thresholds, from 5 × 10^−8^ to increase by an order of magnitude each time until 1. Next, the PRSs were regressed to longevity phenotypes by a logistic regression model. PRSs with smallest *p*‐values were defined as best PRS for each trait.

### 4.4 Definition of the *APOE* region

LD block analysis of *APOE* was performed on our genotype data including 4477 Han Chinese individuals and genotype data of Eastern Asian of the 1000 Genome Project. LDBlockShow (Dong et al., 2021) was used to perform this analysis within the region of chr19:45,311,941–45,512,079, before 100 kb of rs429358 (chr19:45,411,941) and after 100 kb of rs7412 (chr19:45,412,079).

### 4.5 Construction of stratified PRSs and regressed them to longevity

We applied stratified PRSs of SCZ and T2D through two strategies: (1) Cell‐type partitioning; (2) *p*‐value thresholding. Firstly, we carried out the stratified PRSs by partitioning all SNPs into 220 cell types and 10 cell‐type groups then regressed the stratified PRSs to longevity phenotypes. Cell‐type and cell‐type group‐specific annotations for each SNP were obtained from the study of LD score regression (Finucane et al., 2015). In which, the cell‐type annotations for histone modifications (H3K4me1, H3K4me3, H3K9ac, and H3K27ac) were sorted out based on the Roadmap Epigenomics Project (Roadmap Epigenomics Consortium, 2015). Secondly, we constructed stratified PRSs by dividing all SNPs into 8 SNP sets, the *p*‐value thresholds as 5 × 10^−8^, 1 × 10^−7^, 1 × 10^−6^, 1 × 10^−5^, 1 × 10^−4^, 1 × 10^−3^, 1 × 10^−2^, 5 × 10^−2^. To apply the stratified PRS construction, we used a set‐based clumping and thresholding method with the default parameters in PRSet (Choi & O'Reilly, 2019). After stratified PRS construction, we regressed the set‐based PRSs to longevity. We assessed statistical significance at *p* < 0.05 after FDR corrections for 220 × 16 = 3520 tests, the numbers represent 220 cell types and top 16 complex phenotypes whose PRSs were most significantly associated with longevity.

### 4.6 Dissecting effects of pleiotropic SNPs

In order to explore the function of SNPs which both influence longevity and complex diseases, we analyzed the distributions of SNPs' effect size between longevity and complex diseases. We selected the SNPs that were used in the construction of the best PRSs and then selected the SNPs that with *p*‐value < 0.05 in both longevity and complex traits/diseases to plot the effect size distributions. We transformed the odds ratio (OR) to beta value, $beta=\log\left(OR\right)$. All effect alleles recorded in the T2D GWAS summary statistics for the trans‐ethnic T2D GWAS meta‐analysis as published in Mahajan et al. were alleles that increased the risk of T2D. For the SCZ summary statistics, we consolidated all the effect alleles into risk alleles, the beta of effect allele is equal to the negative of beta of non‐effect allele.

### 4.7 Gene annotation and functional pathway enrichment

SNPs used in the best PRS construction were extracted and annotated by ANNOVAR (Yang & Wang, 2015). The annotated genes entered GO enrichment analyses in DAVID (Huang da et al., 2009a; Huang da et al., 2009b). Different panels of genes were classified into three GO classes, Biological Process, Cellular Component, and Molecular Function, respectively. We defined the significant enrichments as those *p*‐value < 2.22 × 10^−4^ (*p* < 0.05/225), after Bonferroni correction.

### 4.8 Longevity predicting model construction using all PRSs

The prediction of longevity can be portrayed as a binary classification problem. Generally, classification algorithms can output a Bernoulli distribution for each sample and choose the label with higher possibility as its prediction. To pursue the optimized regression model, the following steps had been established: (1) Classifier selection. We tried various classifiers, including support vector machine (SVM), k‐nearest neighbors (KNN), Naive Bayes, logistic regression, Decision Tree, and Random Forest classifiers, and selected the best method with ROC (receiver operating characteristic) curves and mean accuracy scores; (2) hyper‐parameters tuning. Appropriate hyper‐parameters were acquired with the grid‐search method; (3) repeated k‐fold cross‐validation. We evaluated the optimum model from adequate randomly generated training and validation subsets. Scikit‐learn (Pedregosa et al., 2011) was used primarily. Consequently, we fixed the logistic regression classifier in the further experiment.

To avoid model overfitting, regularization techniques were applied to train our models. Due to the limitations of scikit‐learn, we can only apply the approaches of L1 and L2 penalties. To find the optimized training hyper‐parameters, we used the scikit‐learn built‐in grid‐search method to tune the hyper‐parameters of the logistic classifier (Pedregosa et al., 2011). The grid‐search method exhaustively considered every possible combination of the parameters and trained the data with each option, then selected the best model available. In this experiment, solver to use in the model, regularization strength, and training iteration numbers provided by scikit‐learn were utilized in our grid‐search. We used the “saga” solver, an implementation of the Stochastic Average Gradient method, with L2 penalties. A larger regularization strength had also been settled for better accuracy. We used the 10‐fold cross‐validation and repeated the whole process for 100 iterations. The model hyper‐parameters with the best accuracy on the given valid datasets and labels were picked out. Finally, we trained the model again with the hyper‐parameters obtained on the 80% training dataset and tested it against the 20% test dataset.

## AUTHOR CONTRIBUTIONS

Yan Li, Yi Zeng, and Chao Nie designed the study. Dingxue Hu and Detao Zhang analyzed the data and wrote the draft manuscript. Dingxue Hu, Yan Li, and Jiahong Ding revised the manuscript. Dingxue Hu and Yan Li reviewed and edited the manuscript. Chao Nie and Yi Zeng provided the materials and data for this study. All authors read and approved the final version of the manuscript.

## CONFLICT OF INTERESTS

None declared.

## Supporting information


Figures S1–S4

Figure 1

Figure 2

Figure 3

Figure 4

## Data Availability

All data requests should be submitted to the corresponding author for consideration. Access to anonymized data might be granted following investigator review.
