# Supplementary material for: Genetic trade‐offs between complex diseases and longevity
Source: Aging Cell. 2022 Jun 26;21(7):e13654. doi: 10.1111/acel.13654 (PMC9282840; doi:10.1111/acel.13654)
Supplement: Supplementary file 1 — Figures S1–S4 [file ACEL-21-e13654-s002.docx]

## Supplementary Materials:

Supplementary Tables were available at the site: <https://figshare.com/s/3014faee2d833b2f44bf>


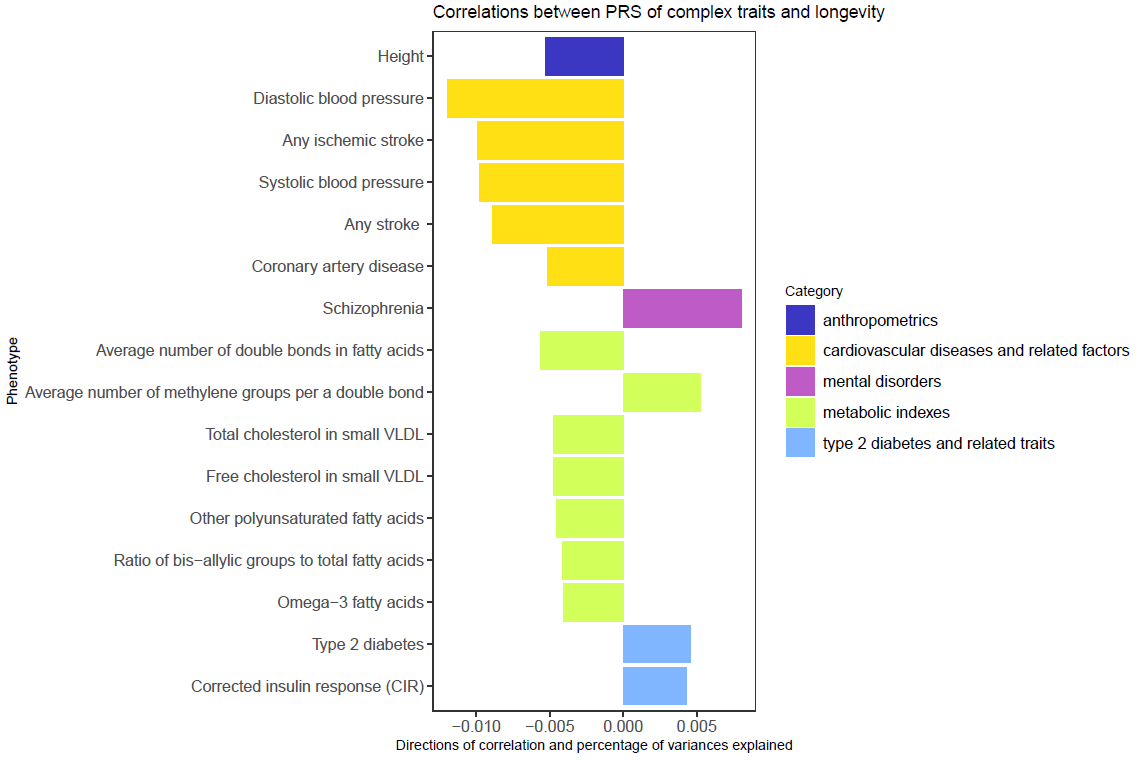


Supplementary Figure1 16 PRSs of complex traits could predict longevity significantly. The length of the bar represents the proportion of longevity explained by PRS. The minus sign indicates negative correlation.


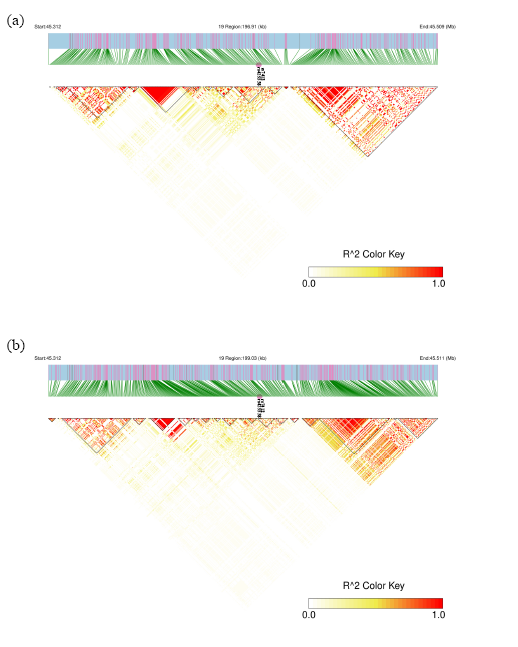


Supplementary Figure 2 LD region of *APOE*

(a): LD block analysis for the 2,178 centenarians and 2,299 middle-aged individuals, 8 LD blocks were in the *APOE* region, as SNPs located form positions 45,361,224 to 45,432,557 base pairs on chromosome 19. (b): LD block analysis for the Eastern Asian population of the 1,000 Genome Project, 14 LD blocks were in the *APOE* region, as SNPs located form positions 45,361,224 to 45,436,657 base pairs on chromosome 19.


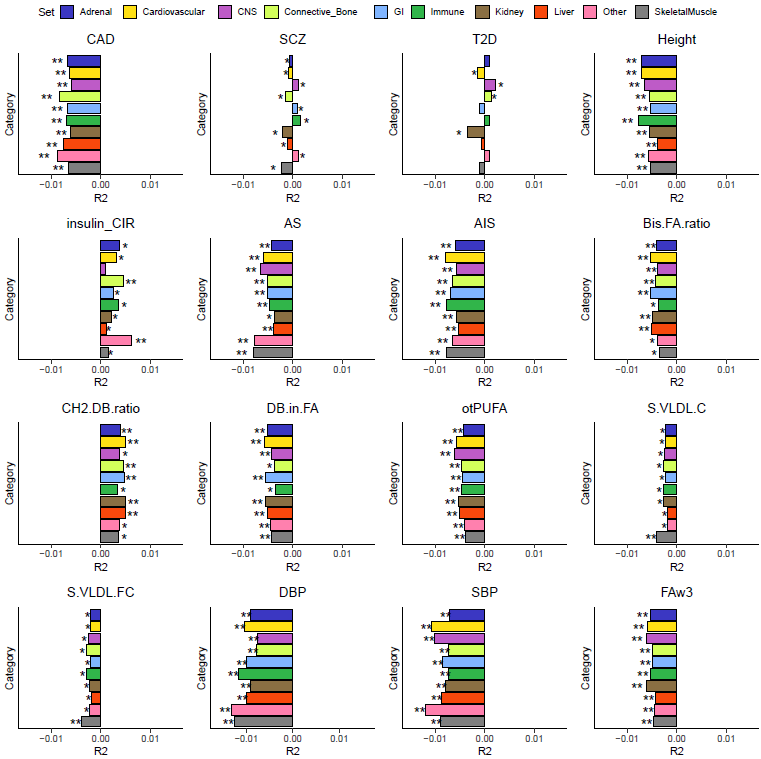


Supplementary Figure 3 Correlations between cell-type group specific PRSs of complex traits and longevity without *APOE* region

The length of the bar represents the proportion of longevity explained by cell-type group specific PRS. The minus sign indicates negative correlation. Phenotype abbreviations were given in Supplementary Table 1.

*: FDR-adjusted p < 0.05

**: p < 0.05 after Bonferroni correction


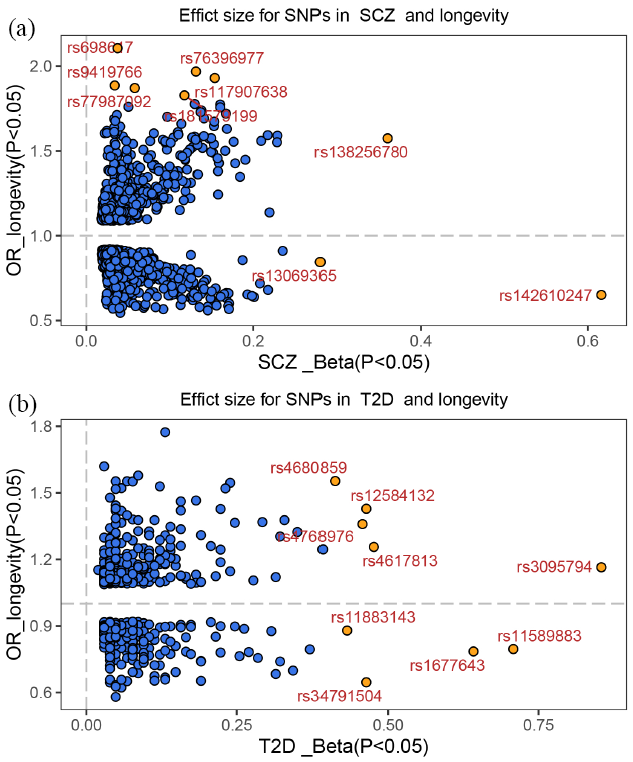


Supplementary Figure 4 Distribution of effect size for SNPs in SCZ, T2D and longevity. Panel 1: SNPs above the horizontal line, both increasing chance of SCZ and longevity; Panel 2: SNPs under the horizontal line, increasing SCZ risk and reducing the chance of longevity.
