# Supplementary figures and images for: Genetic trade‐offs between complex diseases and longevity
Source: Aging Cell. 2022 Jun 26;21(7):e13654. doi: 10.1111/acel.13654 (PMC9282840; doi:10.1111/acel.13654)

Correlations between PRS of complex traits and longevity

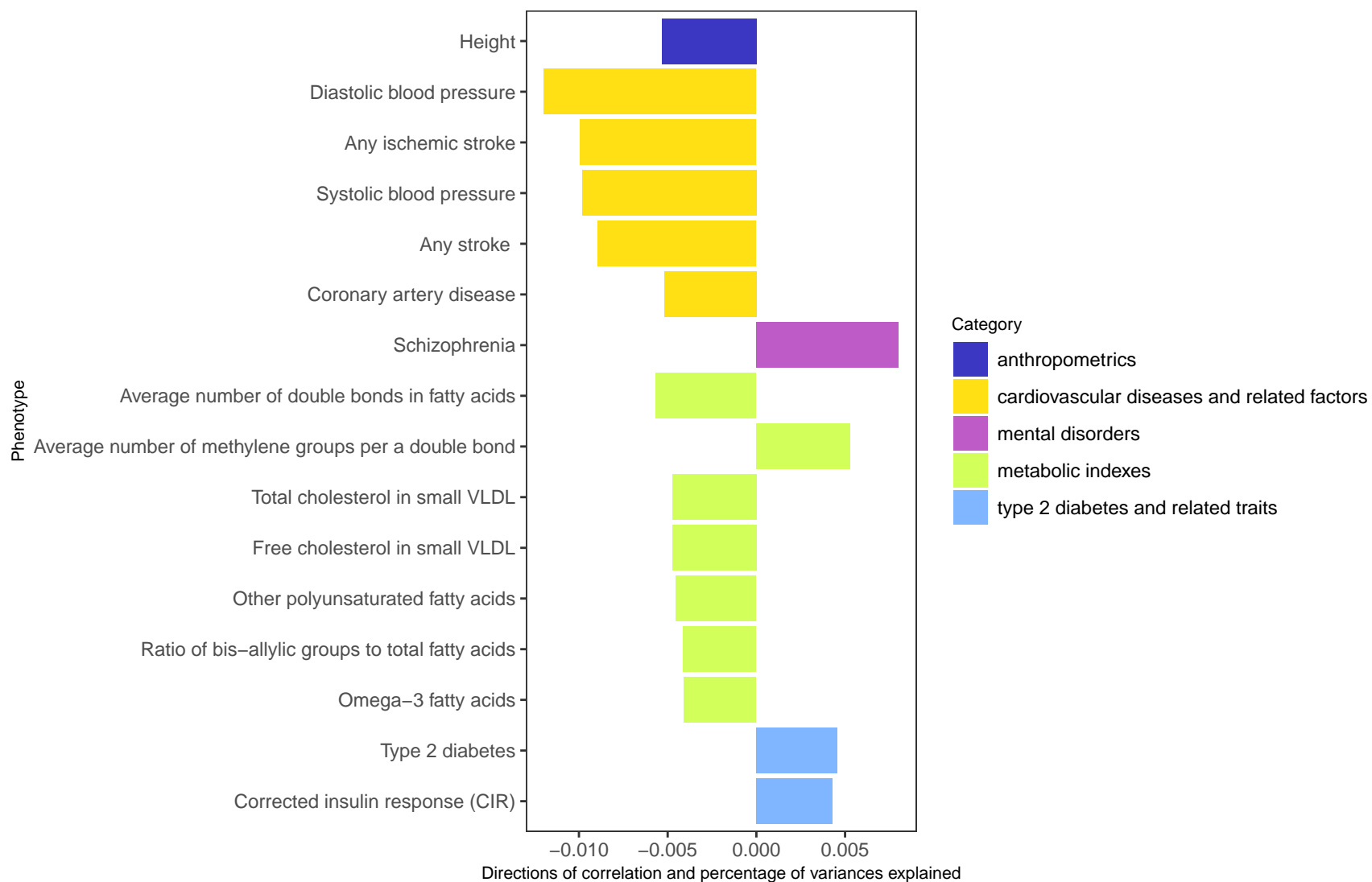

Supplement: Supplementary file 2 — Figure 1 [file ACEL-21-e13654-s001.pdf]

(a)

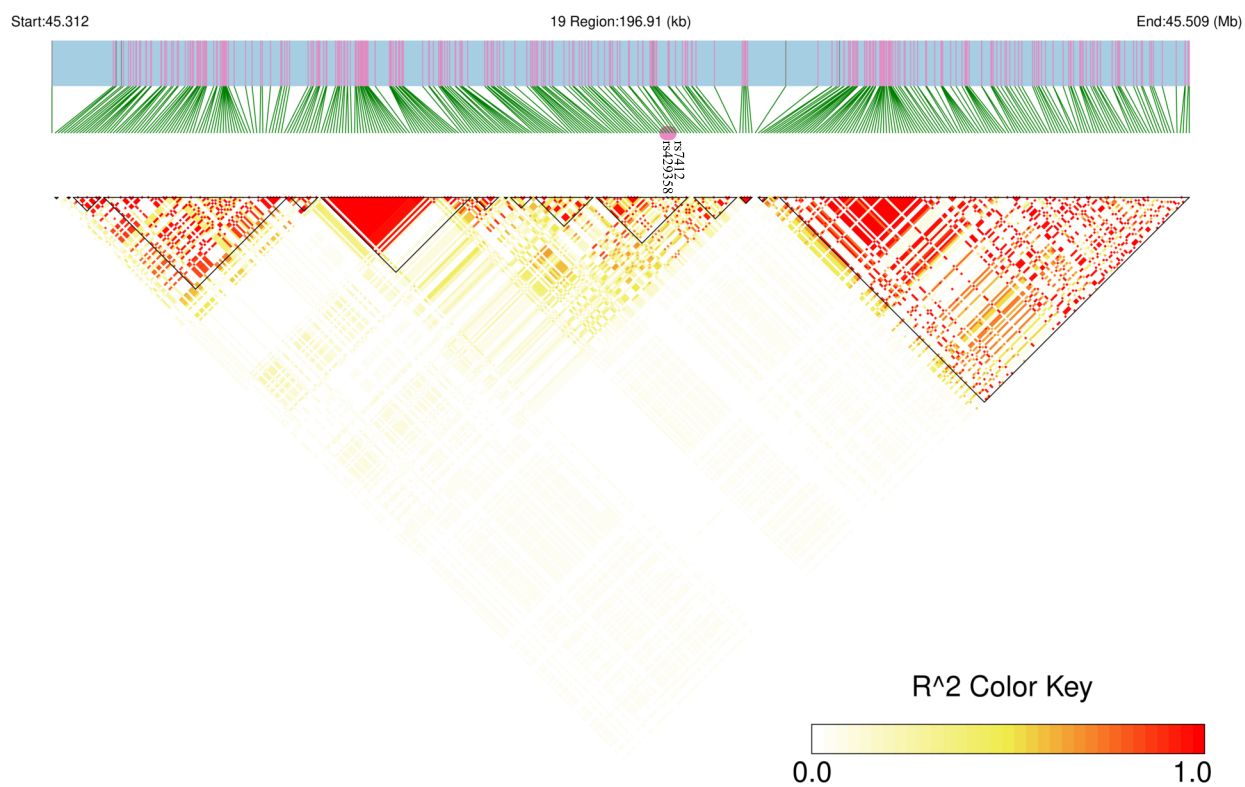

(b)

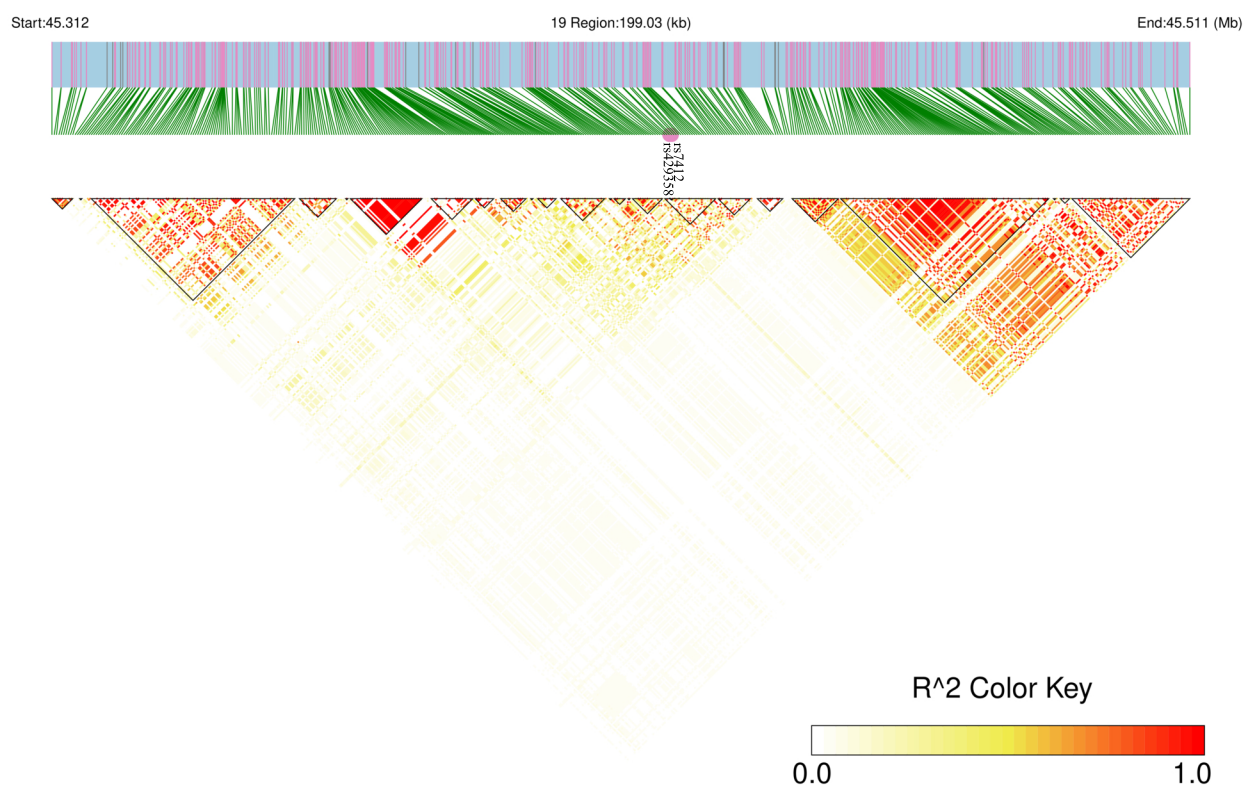

Supplement: Supplementary file 3 — Figure 2 [file ACEL-21-e13654-s003.pdf]

(a) Effect size for SNPs in SCZ and longevity

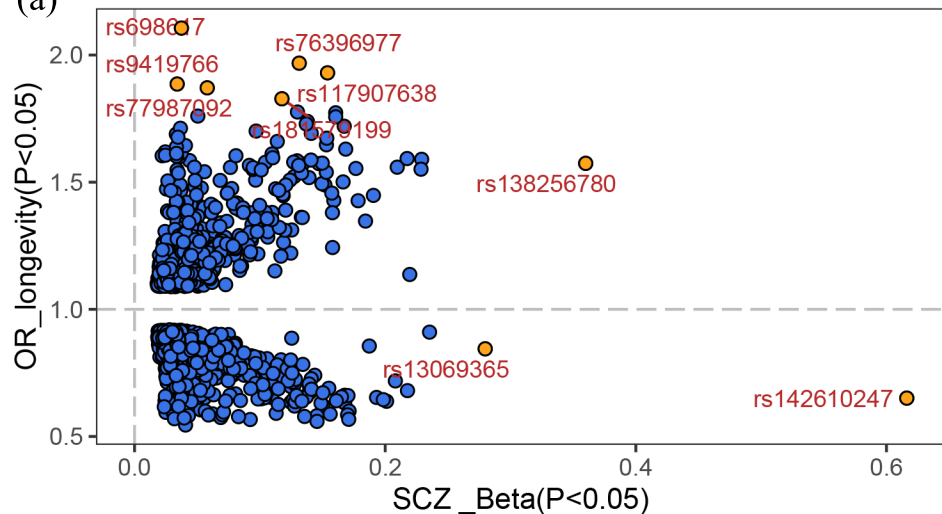

(b) Effect size for SNPs in T2D and longevity

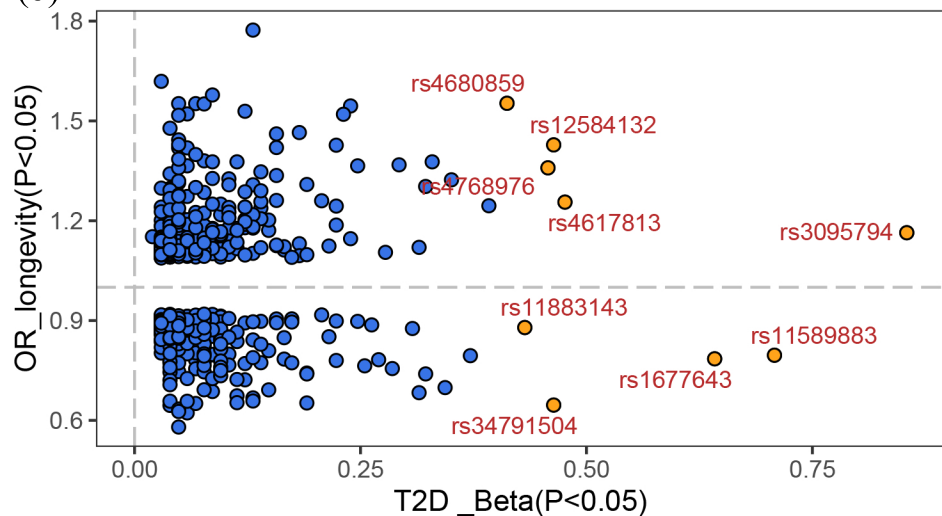

Supplement: Supplementary file 5 — Figure 4 [file ACEL-21-e13654-s004.pdf]
