# Supplementary material for: Genetic trade‐offs between complex diseases and longevity
Source: Aging Cell. 2022 Jun 26;21(7):e13654. doi: 10.1111/acel.13654 (PMC9282840; doi:10.1111/acel.13654)
Supplement: Supplementary file 4 — Figure 3 [file ACEL-21-e13654-s005.pdf]

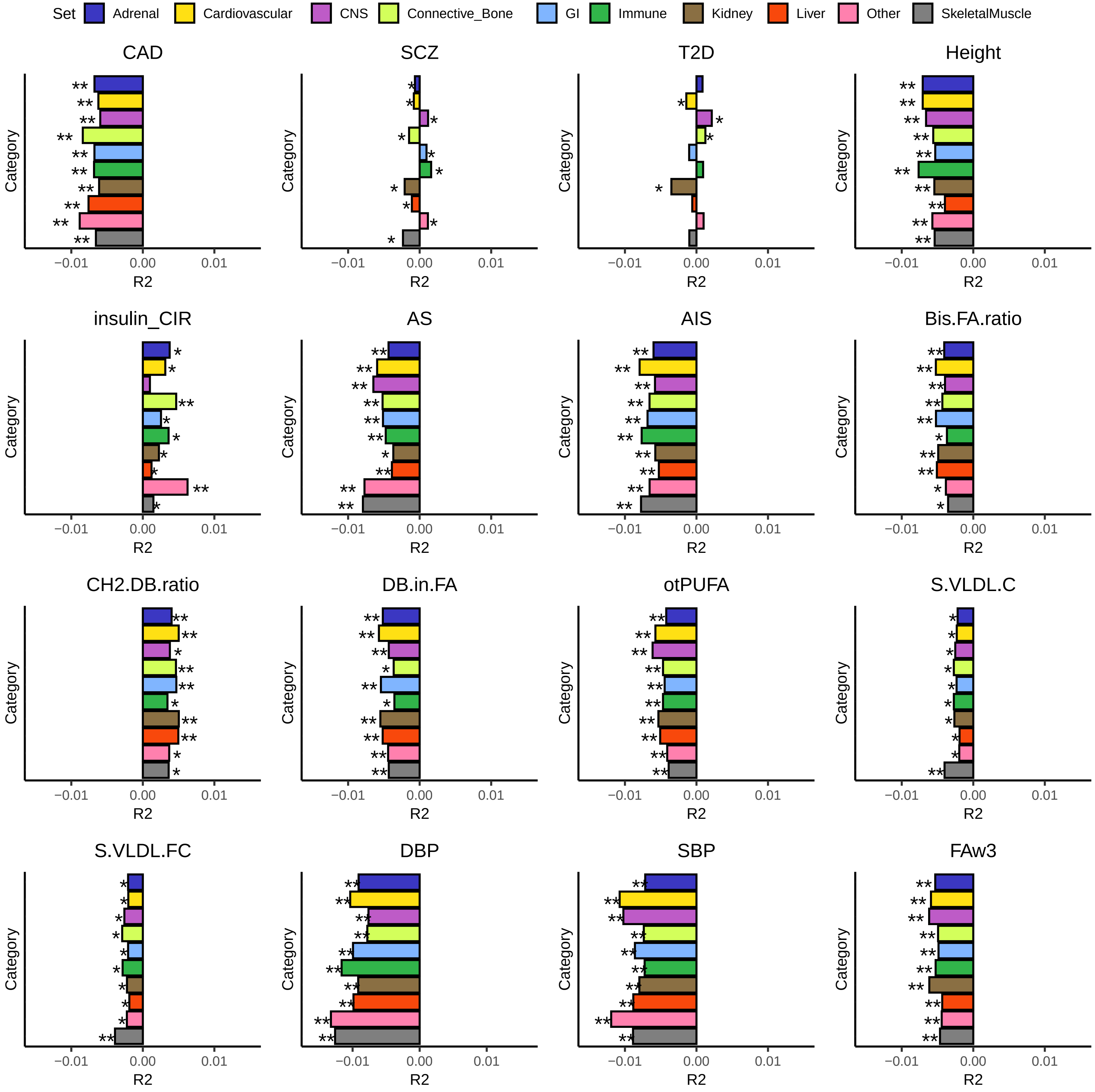

Set
Adrenal
Cardiovascular
CNS
Connective_Bone
GI
Immune
Kidney
Liver
Other
SkeletalMuscle
CAD
SCZ
T2D
Height
insulin_CIR
AS
AIS
Bis.FA.ratio
CH2.DB.ratio
DB.in.FA
otPUFA
S.VLDL.C
S.VLDL.FC
DBP
SBP
FAw3
Category
R2
−0.01
0.00
0.01
